# Peripheral and central effects of NADPH oxidase inhibitor, mitoapocynin, in a rat model of diisopropylfluorophosphate (DFP) toxicity

**DOI:** 10.3389/fncel.2023.1195843

**Published:** 2023-06-21

**Authors:** Christina Meyer, Nikhil S. Rao, Suraj S. Vasanthi, Beatriz Pereira, Meghan Gage, Marson Putra, Claire Holtkamp, Joselyn Huss, Thimmasettappa Thippeswamy

**Affiliations:** Department of Biomedical Sciences, College of Veterinary Medicine, Iowa State University, Ames, IA, United States

**Keywords:** oxidative stress, proinflammatory cytokines, diisopropylfluorophosphate (DFP), GP91*^phox^*, gliosis

## Abstract

Organophosphates (OP) are highly toxic chemical nerve agents that have been used in chemical warfare. Currently, there are no effective medical countermeasures (MCMs) that mitigate the chronic effects of OP exposure. Oxidative stress is a key mechanism underlying OP-induced cell death and inflammation in the peripheral and central nervous systems and is not mitigated by the available MCMs. NADPH oxidase (NOX) is one of the leading producers of reactive oxygen species (ROS) following *status epilepticus* (SE). In this study, we tested the efficacy of the mitochondrial-targeted NOX inhibitor, mitoapocynin (MPO) (10 mg/kg, oral), in a rat diisopropylfluorophosphate (DFP) model of OP toxicity. In DFP-exposed animals, MPO decreased oxidative stress markers nitrite, ROS, and GSSG in the serum. Additionally, MPO significantly reduced proinflammatory cytokines IL-1β, IL-6, and TNF-α post-DFP exposure. There was a significant increase in GP91*^phox^*, a NOX2 subunit, in the brains of DFP-exposed animals 1-week post-challenge. However, MPO treatment did not affect NOX2 expression in the brain. Neurodegeneration (NeuN and FJB) and gliosis [microglia (IBA1 and CD68), and astroglia (GFAP and C3)] quantification revealed a significant increase in neurodegeneration and gliosis after DFP-exposure. A marginal reduction in microglial cells and C3 colocalization with GFAP in DFP + MPO was observed. The MPO dosing regimen used in this study at 10 mg/kg did not affect microglial CD68 expression, astroglial count, or neurodegeneration. MPO reduced DFP-induced oxidative stress and inflammation markers in the serum but only marginally mitigated the effects in the brain. Dose optimization studies are required to determine the effective dose of MPO to mitigate DFP-induced changes in the brain.

## Introduction

Chemical nerve agents (CNAs) are a unique class of highly lethal neurotoxins and historically exploited in chemical warfare (Diauudin et al., 2019). Originating as a pesticide, organophosphate (OP) compounds were first synthesized as potent CNAs in the early 1930s, just in time for World War II, and have since been employed in the Persian Gulf War, Syria conflict, Matsumoto and Tokyo terrorist attacks, and political assassinations (Gupta, 2015; Chai et al., 2017). OPs, including tabun, sarin, soman, novichok, and diisopropylfluorophosphate (DFP), irreversibly inhibit acetylcholinesterase (AChE), an enzyme responsible for the breakdown of acetylcholine (ACh). The inhibition of AChE results in the accumulation of ACh, promoting acute muscarinic and nicotinic hyperexcitability in both peripheral and central nervous systems leading to respiratory depression, muscle fasciculation, status epilepticus (SE), and in extreme cases, death (Marrs, 1993; Holstege et al., 1997; Franca et al., 2019). Long-term consequences can be severe, resulting in neurodegeneration, neuroinflammation, behavioral deficits, and spontaneous recurrent seizures, i.e., epilepsy (Okumura et al., 1996; Miyaki et al., 2005).

The currently available medical countermeasures (MCMs) for OP toxicity control acute symptoms but are ineffective for SE-induced brain injury. While select military personnel carry MCMs on the field, it is unlikely that civilians will have early access in an exposure scenario. FDA-approved treatments for OP exposure include the cholinergic antagonist, atropine, the cholinesterase reactivator, 2-pralidoxime chloride (2-PAM), and the GABA allosteric modulator, Midazolam (Newmark, 2019). Atropine and 2-PAM can effectively reduce mortality by mitigating peripheral, but not central, effects of OP exposure; therefore, they do not offer long-term protection (Gao et al., 2016; Spampanato et al., 2019). Midazolam has been shown to terminate OP-induced behavioral SE following acute exposure, however, its neuroprotective effect depends on how quickly the drug is administered (Supasai et al., 2020). Moreover, the currently available MCMs fail to mitigate OP-induced oxidative stress, a critical underlying mechanism of neuroinflammation, neurodegeneration, and seizures that follow SE (Chang and Yu, 2010; Puttachary et al., 2015; Guignet et al., 2020).

Oxidative stress, or the imbalance between the generation of oxygen free radicals and antioxidants, is a consequence of OP intoxication in humans and rodents alike (Ranjbar et al., 2005; Pearson and Patel, 2016). Oxygen free radicals are oxygen molecules with an unpaired electron. They are highly reactive, causing lipid peroxidation, protein degradation, and DNA damage in their attempt to “steal” electrons (Lukaszewicz-Hussain, 2010; Walker, 2023). In the periphery, excess reactive oxygen species (ROS) increase proinflammatory cytokine production (Chen et al., 2018). In *in vitro* models of OP toxicity, oxidative stress was associated with lymphocytic death, nephrotoxicity, and gut dysbiosis (Gao et al., 2016; Ahmad et al., 2019; Sobolev et al., 2022; Gage et al., 2023). The central nervous system is especially vulnerable to ROS due to the brain’s high oxygen metabolic rate and concentration of polyunsaturated fatty acids (Vanova et al., 2018; Lee et al., 2021). The neuroinflammatory response to acute OP exposure, including astrogliosis and microgliosis, along with neuronal excitation, likely drives the initial production of ROS (Badr, 2020; Lee et al., 2021). Increased levels of ROS, gliosis, and neuronal loss create a chronic excitotoxic environment in the brain that lowers the seizure threshold and spurs cognitive and memory dysfunction (Chen, 2012).

Although mitochondria are traditionally considered the primary contributors to oxidative stress because of high ATP consumption during OP-induced SE (Milatovic et al., 2006; Lukaszewicz-Hussain, 2010), emerging evidence suggests the NADPH oxidase (NOX) is also a significant primary producer of free radicals (Walker, 2023). NOX activation creates a feed-forward mechanism of ROS generation in concurrence with the mitochondria (Dikalov, 2011). Thus, both NOX and mitochondria are highly implicated in oxidative stress. Increased concentrations of the oxidative stress markers ROS, oxidized glutathione (GSSG), nitrite, and the NOX2 subunit GP91*^phox^*, support mitochondrial dysfunction and NOX upregulation following OP exposure (Mostafalou and Abdollahi, 2018; Putra et al., 2020a). Therefore, targeting NOX activity can be an option for attenuating OP toxicity-induced oxidative stress.

Apocynin is a broad NOX inhibitor, though it has been suggested to have an affinity for NOX2 inhibition (Weston et al., 2013). NOX2 is one of seven in the NOX family, and is expressed in phagocytic cells such as microglia and abundantly upregulated in neurological disorders (Sumimoto et al., 2005). Furthermore, apocynin has been found to reduce mitochondrial dysfunction (Du et al., 2019). In neurodegenerative disease models such as Parkinson’s Disease and epilepsy, as well as diabetic cardiomyopathy and hypertension, apocynin reduced neuronal death, microglial and astroglial activation, inflammation, and excessive ROS production (Costa et al., 2009; Roe et al., 2011; Ghosh et al., 2012; Jaiswal and Kumar, 2022). Previously, we discovered that diapocynin (DPO), a dimer of apocynin, suppressed astrogliosis, neurodegeneration, and GP91*^phox^* expression in rat brains 6 weeks post-DFP exposure (Putra et al., 2020a). Due to their low bioavailability and high dosages required for efficacy (Putra et al., 2020a; Savla et al., 2021), apocynin and DPO are unlikely to be feasible for human use. To circumvent this problem, we tested mitoapocynin (MPO, apocynin TPP^+^ conjugated to enable mitochondrial uptake), which has shown excellent efficacy at a very low dose (3 mg/kg, oral) in a mouse Parkinson’s disease model (Ghosh et al., 2016; Langley et al., 2017). Notably, MPO (10 mg/kg, oral) reduced brain oxidative stress in rats after repeated exposure to a pesticide OP, chlorpyrifos (Singh et al., 2018). We predicted that treatment with 10 mg/kg MPO (oral) will also reduce oxidative stress markers in the peripheral and central nervous systems in response to acute exposure to DFP.

## Materials and methods

### Animal source and care

A mixed-sex cohort of adult Sprague Dawley rats (7–8 weeks; *N* = 40) was purchased from Charles River Laboratories (MA, USA). Upon arrival, animals were habituated for 3 days before handling. Males and females were individually housed in the same room (19–23°C, 12–h light/dark cycles) with free access to food and water at the Laboratory of Animal Recourses, Iowa State University (ISU). DFP challenge, drug dosing, and euthanasia procedures were approved by the ISU Institutional Care and Use Committee. All experiments complied with NIH ARRIVE guidelines.

### Chemicals

DFP (97.8% pure, Sigma-Aldrich) was prepared fresh in cold 0.1M phosphate-buffered saline (PBS). We prepared both atropine sulfate (ATS, Tokyo Chemical Industry) and 2-PAM (Sigma) fresh in saline. Midazolam (MDZ) and pentobarbital sodium were purchased from the Lloyd Veterinary Medical Center pharmacy at ISU. MPO (American Biochemicals, TX) was dissolved in 100% ethanol then diluted in sterile dH2O for a final vehicle of 2% ethanol. LC-MS determined the purity of MPO in 100% ethanol to be 99.9%. A 4% paraformaldehyde (PFA, Acros Organics) in PBS solution was made and then stored at 4°C until it was used for animal perfusion. The primary antibodies used in immunohistochemistry (IHC) were NeuN (rabbit polyclonal, 1:200, EMD Millipore), IBA1 (goat polyclonal, 1:300, Abcam), GP91*^phox^* (mouse monoclonal, 1:100, Santa Cruz Biotechnology), CD68 (Rabbit polyclonal, 1:400, Abcam), C3 (rabbit monoclonal, 1:60, Novus Biologicals), and GFAP (mouse monoclonal, 1:300, Sigma). The secondary antibodies Alexa Flour 488 anti-mouse and anti-rabbit (1:200, 1:80, respectfully), Rhodamine red X anti-rabbit (1:200), Biotin anti-goat (1:300), and Streptavidin cy3 (1:200) were purchased from Jackson ImmunoResearch. Colocalization of Fluoro-jade B (FJB, Histochem) and NeuN was quantified for a neurodegeneration measure.

### DFP exposure, SE scoring, and MPO administration

**FIGURE 1:**
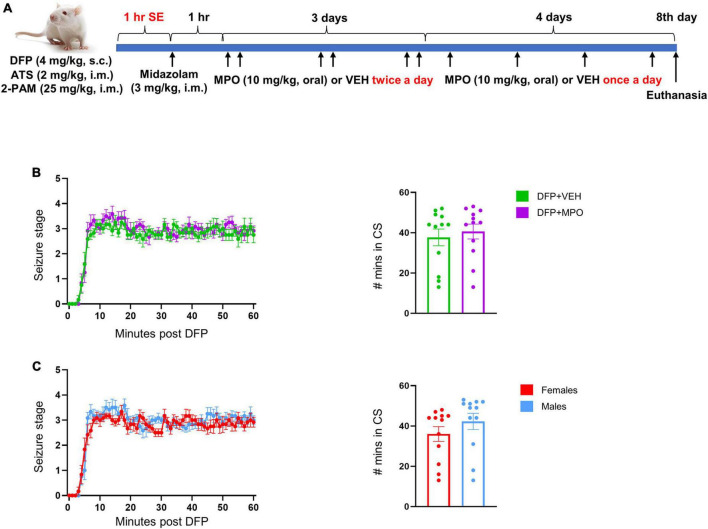
A summary of experimental design and SE quantification. **(A)** Schematic illustration of the experimental timeline of DFP administration and MPO treatment. **(B,C)** SE severity by seizure stage over time and SE severity score- the time (in minutes) spent in CS (≥stage 3) during the 60 min post DFP exposure in-mixed sex cohort **(B)** and between males and females **(C)**. There was no significant difference in SE severity between vehicle and MPO treated animals (**B**, mixed effects analysis) or between males and females (**C**, unpaired *t*-test). *n* = 12 per group. Data presented as mean ± SEM.

DFP (4 mg/kg, s.c.) challenged animals were split into two randomized batches, 12 animals each (6 per sex). We chose the DFP dose based on our previous studies testing different concentrations to achieve mild, moderate, and severe seizures in males and females (Putra et al., 2020b; Rao et al., 2022). As illustrated in Figure 1A, the rats were immediately (<1 min) given ATS (2 mg/kg, i.m.) and 2-PAM (25 mg/kg, i.m.) following DFP to mitigate the acute peripheral effects of the irreversible AChE inhibition, and by proxy, mortality. For the following hour, two experimenters scored behavioral seizures based on a modified Racine scale as described in our previous studies (Gage et al., 2022b; Rao et al., 2022). The stages are defined as follows: Stage 1: SLUD (salivation, lacrimation, urination, and defecation); Stage 2: head nodding, wet dog shakes, and mastication; Stage 3: rearing, front limb clonus, and Straub tail; Stage 4: rearing with loss of righting reflex; Stage 5: inability to stand, abducted limbs, or erratic jumping. Stages 1 and 2 are considered non-convulsive seizures, whereas stages ≥ 3 are convulsive seizures (CS). A third experimenter oversaw the two lead scorers to cross-validate their observations.

To terminate behavioral SE, MDZ (3 mg/kg, i.m.) was administered alongside 1 ml of 5% dextrose normal saline (s.c.). Animals were paired based on minutes spent in CS, then randomized to VEH (2% ETOH in dH2O, oral) or MPO (10 mg/kg, oral) and administered an hour after MDZ. Both treatment groups were dosed twice a day for the first 3 days and once a day for the next 4 days of the experiment. The animals were provided moistened food pellets with Nutri-Cal (Vetoquinol) and 5% dextrose saline (1 ml, s.c.) until weight gain was observed.

### Euthanasia

At day 8, all DFP-exposed animals and the age- and sex-matched control animals were euthanized by administering pentobarbital sodium (100 mg/kg, i.p.). Blood was collected by severing the caudal vena cava, and serum was separated and used in ELISA, Griess, ROS, and GSSG assays. Animals were perfused with PBS followed by 4% PFA as in past studies (Gage et al., 2021). A needle was inserted into the left ventricle, near the apex of the heart, to first flush with PBS (5 min), then 4% PFA (15 min) at 60 mL/min, 80 mm Hg.

### Griess (nitrite) assay

A Griess assay (Sigma-Aldrich) was used to estimate the concentration (μM) of nitrite in the serum. In duplicates, 50 μL of undiluted serum and 50 μL of Griess reagent were added to a 96-well plate. Duplicate serial dilutions of sodium nitrite solution were added to the wells to generate a standard curve. Nitrite concentrations were interpolated from the OD values using the standard curve. The plate was then incubated at room temperature (RT) in the dark for 10 min. A Spectramax M2 (Molecular Devices) was used to detect color change at 540 nm absorbance.

### ROS assay

Superoxide anion levels in the serum were detected using a reactive oxygen species assay kit (OxiSelect, USA). The standard curve was established with a 1:10 serial dilution of 7′-dichlorodihydrofluorescin. The samples were added in duplicates, 50 μL per well, and were mixed with 50 μL of Catalyst and incubated for 5 min at RT. At RT, 100 μL of dichlorodihydrofluorescin-DiOxyQ solution was added to each well and incubated for 30 min in the dark. A Spectramax M2 (Molecular devices) was used to read the plates at 480 nm excitation/530 nm emission. The control group fluorescence value was subtracted as background, and the results were reported as relative fluorescence values (RFUs).

### Oxidized glutathione assay

To measure the concentration (μM) of oxidized glutathione (GSSG) in the serum, we used a glutathione colorimetric detection kit (Thermo Fisher, USA). Serum samples were deproteinized with cold 5% 5-sulfo-salicylic acid dehydrate (SSA) 1:1 and incubated at 4°C for 10 min. The samples were then centrifuged at 4°C for 10 min at 14,000 rpm, and the supernatant was collected. The samples were treated with 5 μL of 2-vinylpyridine (2VP) for every 250 μL of sample and incubated at RT for 1 h. A 2VP standard solution was serially diluted to generate a standard curve. For detection, 50 μL of samples or standards were added in duplicates, followed by 25 μL of colorimetric detection reagent into each well. Next, 25 μL of reaction mixture was added to each well, and the plate was tapped to mix. The plate was incubated at RT for 20 min and the absorbance was read immediately by a Spectramax M2 (Molecular Devices) at 405 nm.

### ELISA

ELISA kits (RayBiotech) were used to estimate serum cytokine and chemokine levels (pg/ml). The cytokines that were probed consisted of interleukins (ILs) IL-1β, IL-6, IL-10, tumor necrosis factor-alpha (TNF-α), and monocyte chemoattractant protein-1 (MCP1). Serum samples were added to a 96-well plate in 50 μL duplicates and diluted 1:1 with assay diluent. To establish a standard curve, 10,000 pg/ml standard protein was serially diluted by a factor of 2.5. The plates were then incubated at RT for 2.5 h with gentle shaking. The solution was discarded and washed 4 times with Wash Buffer. Next, 100 μL of biotinylated antibody was added to each well and incubated for 1 h before washing an additional 4 times. The plates were incubated in 100 μL streptavidin per well for 45 min with gentle shaking at RT. After 4 washes, 100 μL of tetramethylbenzidine one-step substrate reagent was added to each well and left at RT with gentle shaking for 30 min in the dark. Finally, 50 μL of Stop Solution was added to each plate and immediately placed in a Spectramax M2 (Molecular Devices) at 480 nm absorbance detection. Cytokine concentrations were interpolated from the OD values using the standard curve.

### Tissue processing, immunohistochemistry, and cell quantification

Brain tissue was post-fixed in 4% PFA for 24 h, then switched to 25% sucrose in PBS for 3 days before embedding in gelatin (15% type A porcine gelatin, 7.5% sucrose, 0.1% sodium azide). Brain tissue was incubated in gelatin at 37°C for 1 h and left overnight at 4°C. Tissue blocks were frozen in 2-methylbutane cooled by liquid nitrogen and stored in a −80°C freezer. Brain tissue blocks were sectioned rostral to caudal (16 μm) using a cryostat (Thermo Fisher) as described in our prior publications (Putra, 2017; Gage et al., 2022a). The slides were stored in a −20°C freezer until they were processed for IHC.

Before IHC, antigen retrieval was performed by placing slides in citric acid buffer (10 mM citric acid, 0.05% Tween 20, pH 6) heated to 95°C for 25 min, then set aside to cool. The slides were loaded onto a Shandon rack and washed with PBS (3 times), and the sections were incubated with 10% donkey serum, 0.05% TritonX-100 in PBS an hour to block non-specific binding sites. After adding the primary antibody to the slides, the Shandon rack was stored at 4°C overnight. The next day, the slides were placed in RT for 20 min, washed with PBS (3 times), then incubated in the secondary antibody (conjugated or biotinylated) for an hour. Tissues treated with biotinylated antibody underwent an additional incubation with streptavidin at RT for an hour. After the final wash with PBS, coverslips were placed on the slides with Vectashield Antifade mounting media containing DAPI.

To measure neurodegeneration, FJB staining was conducted. After NeuN staining, slides were washed once with PBS and then twice with dH2O. The slides were removed from the Shandon rack and immersed in 0.006% KMn0_4_ in dH2O for 5 min. Again, the slides were washed in dH2O before submerging them in 0.0003% FJB for 10 min in the dark and dried at RT in the dark. Finally, the slides were dipped in xylene and mounted with Surgipath Acrytol.

A Leica DMi8 (Thermo Fisher) inverted fluorescent microscope was used to image immunostained brain sections and regions of interest. All images except glial scars were taken at 20X objective Z-stack for 11 captures at 1.8 μm intervals. The regions of interest include hippocampal CA1, CA3, and DG, along with extrahippocampal regions amygdala (AMY) and piriform cortex (PC). These regions are strongly affected by DFP, resulting in an increased presence of neurodegeneration (NeuN, FJB), microgliosis (IBA1, CD68, GP91*^phox^*), and astrogliosis (C3, GFAP), as shown in our previous studies (Putra et al., 2020a; Gage et al., 2021). Thus, we focused our imaging and cell quantification of neurons, microglia, and astroglia, as well as the proteins they express in these cells.

The captured images were exported as Tiff files for further analysis. CellProfiler 4.1.3 was used to quantify neurons and microglia. The typical diameter defined in pixel units (min, max) for neurons was 15–90, whereas microglia were identified as 15–80 (1.3466 threshold smoothing scale for both). For NeuN and IBA1 counting, objects touching the image’s border were discarded. All colocalization and GFAP quantification were performed using ImageJ software multi-point tool. Cells positive for DAPI or colocalization were quantified from at least 2 sections (3.5 average) per animal.

### Statistics and methodological rigor

To reduce bias, experimenters were blinded to group assignment, and all the animals were randomized and coded. Behavioral seizure scoring of SE was cross verified by a third experimenter. GraphPad Prism 9.0 was used for all statistical analyses and graphs. The Shapiro–Wilk test for normality was performed to determine the appropriate parametric or non-parametric tests for each analysis. Specific statistical tests for individual analysis can be found in the figure legends. Depending on the number of groups, an unpaired *t*-test or an ANOVA was conducted between normal (parametric) samples. The rank sum-based Mann–Whitney and Kruskal–Wallis test was used for non-parametric data. Statistical significance was considered as *p* ≤ 0.05.

## Results

Experimental design is illustrated in Figure 1A.

### A comparison of DFP-induced SE between vehicle and MPO-treated groups

SE severity directly correlates with brain pathology (Gage et al., 2021; Rao et al., 2022); therefore, to ensure balanced SE severity between treatment groups, we quantified the number of minutes that the animals spent in CS (≥stage 3), and the pattern of seizure stages over time, determined by the modified Racine scale. There was no significant difference in SE severity (Figure 1B) between DFP + VEH and DFP + MPO nor between males and females (Figure 1C) in the seizure stage progression during SE. SE severity is defined as the total duration animals spent in CS between DFP exposure and midazolam treatment, which is ∼60 min (Figures 1B, C).

### MPO suppresses DFP-induced oxidative stress markers in the serum

**FIGURE 2:**
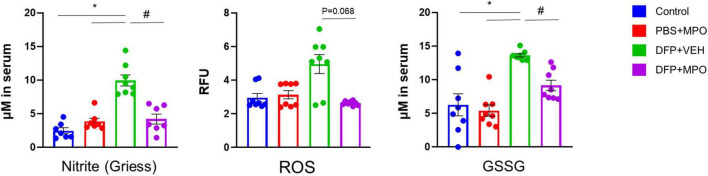
Oxidative stress markers in the serum. Comparison between control, PBS + MPO, DFP + VEH, and DFP + MPO for nitrite (μM) using a Griess assay, superoxide anion levels (RFU) using a reactive oxygen species (ROS) assay, and oxidized glutathione (μM) using a GSSG assay. Compared to the control, there was a significant increase in nitrite and GSSG in DFP + VEH animals, but not in DFP + MPO was observed. DFP + VEH ROS concentration was high (*p* = 0.68) compared to all other groups. Kruskal–Wallis test for nitrite and ROS; and one-way ANOVA for GSSG. *n* = 8 per group. *Represents the DFP effect compared to control and PBS + MPO. #Represents effects of MPO in DFP compared to DFP + VEH. *p < 0.05, #*p* < 0.05. Data presented as mean ± SEM.

To evaluate the presence of oxidative and nitro-oxidative stress markers in the periphery, we quantified the concentration of ROS (RFU), nitrite (μM), and GSSG (μM) in the serum (Figure 2). There was a significant increase in the production of nitrite in the serum of DFP + VEH rats as compared to control groups. MPO significantly reduced the concentrations of serum nitrite a week after the DFP challenge, 24 h after the last dose of MPO (Figure 2). DFP exposure led to an increase of ROS in vehicle-treated animals, which trended toward attenuation in the MPO-treated group, although the difference did not reach statistical significance. GSSG concentration followed the same pattern: a significant increase of GSSG in DFP + VEH and a significant reduction in DFP + MPO (Figure 2).

### MPO suppresses DFP-induced inflammatory markers in the serum

**FIGURE 3:**
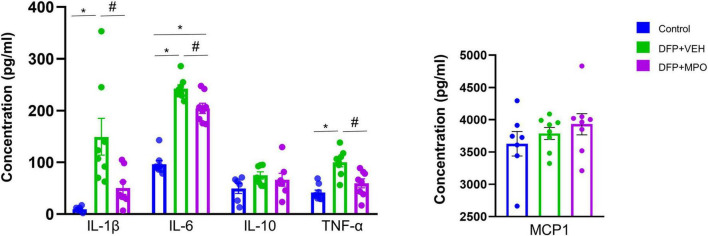
Serum cytokine and chemokine assays. MPO significantly reduced DFP-induced proinflammatory cytokines IL-1β, IL-6, TNF-α, IL-10, and MCP1 levels did not alter in any groups. Kruskal–Wallis test for IL-1β and IL-6 and one-way ANOVA for IL-10, TNF-α, and MCP1. *n* = 8 per group. *Represents the DFP effect compared to control and PBS + MPO. #Represents effects of MPO in DFP compared to DFP + VEH, *p < 0.05, #*p* < 0.05. Data presented as mean ± SEM.

The cytokine and chemokine response was investigated in the serum of DFP-challenged animals and compared with controls. The concentrations (pg/ml) of proinflammatory cytokines IL-1β, IL-6, TNF-α, anti-inflammatory cytokine IL-10, and chemokine MCP1were tested (Figure 3). MPO treatment significantly reduced the concentrations of key proinflammatory cytokines IL-1β, IL-6, and TNF-α in the serum 1-week post-DFP exposure. The cytokine IL-10 and chemokine MCP1 concentrations did not significantly differ across conditions (Figure 3).

### The impact of MPO treatment on DFP-induced GP91*^phox^* expression and microgliosis

**FIGURE 4:**
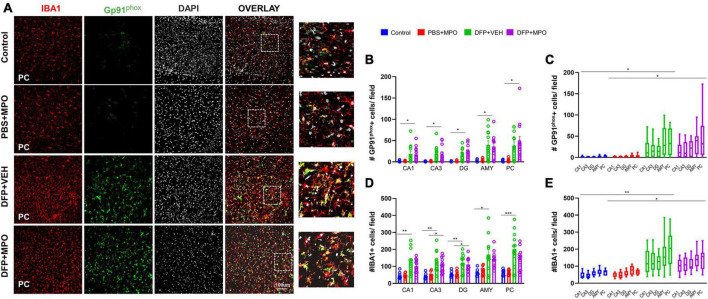
Oxidative stress markers in the brain 1-week post-DFP. **(A)** Representative images of the piriform cortex (PC) showing GP91*^phox^* (green), IBA1-positive microglial cells (red), and DAPI (white), a nuclear stain. The overlay images exemplify colocalization. **(B)** GP91*^phox^* and IBA1 colocalized-microglial cells quantification from CA1, CA3, DG, amygdala (AMY), and PC regions increased in DFP groups compared to non-DFP groups. MPO did not have an effect. DFP + MPO Gp91*^phox^*-positive microglia were significantly higher than control animals in all regions quantified. Kruskal–Wallis test. **(C,E)** The overall group effect of MPO on GP91*^phox^*-IBA1 colocalization and IBA1 positive microglia cells per field in CA1, CA3, DG, Amy, and PC are shown. Horizontal bars indicate a significant difference between control vs. DFP + VEH and PBS + MPO vs. DFP + MPO. Overall, MPO, did not have an effect on GP91*^phox^* IBA1 colocalization. Microglia absolute count was reduced, though it was not significant. Two-way ANOVA mixed effects analysis. **(D)** IBA1-positive microglia was not affected by treatment with MPO. In each region, DFP significantly increased the quantity of IBA1-positive cells. There were significantly more IBA1-positive cells in the CA3 and DG regions of DFP + MPO compared to PBS + MPO animals which further demonstrates the effect of DFP. One-way ANOVA. *n* = 8 control groups, *n* = 12 DFP groups. *p < 0.05, **p < 0.01, ***p < 0.001.

Previously, we demonstrated an upregulation of NOX2 subunit GP91*^phox^* in microglial cells in a SE-induced DFP model (Putra et al., 2020a; Gage et al., 2022b). One week post-DFP challenge, we also found an increased number of GP91*^phox^* positive cells, represented in Figures 4A–C. We observed a significant increase of GP91*^phox^* positive microglial cells in DFP + VEH and DFP + MPO groups compared to control and PBS + NPO groups in the hippocampus (CA1, CA3, DG), AMY, and PC (Figure 4C). In the same brain regions, there was a significant group effect in GP91*^phox^* IBA1 colocalization of DFP groups compared to PBS + MPO animals. However, MPO did not significantly affect the number of GP91*^phox^*-positive microglial cells across the hippocampal and extra-hippocampal regions (Figures 4B, C).

**FIGURE 5:**
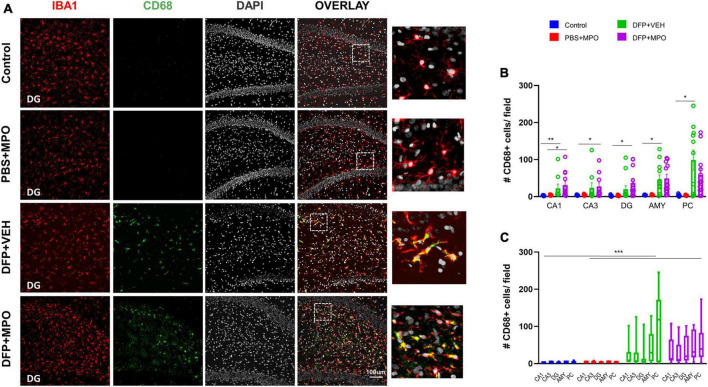
Microgliosis 1-week post-DFP. **(A)** Representative images of CD68 positive (green) microglia (IBA1, red) with DAPI (white) in the dentate gyrus (DG) from controls and DFP-exposed animals treated with vehicle or MPO. **(B)** Quantification of CD68 + microglia from CA1, CA3, DG, amygdala (AMY), and piriform cortex (PC). CD68 expression in DFP groups was upregulated in all these regions compared to controls. Kruskal–Wallis test. **(C)** Bars indicate a significant difference in group effect between control vs. DFP + VEH and PBS + MPO vs. DFP + MPO. Two-way ANOVA mixed effect analysis. *n* = 8 in non-DFP groups, *n* = 12 in DFP groups. *p < 0.05, **p < 0.01, ***p < 0.001.

Indicators of microgliosis include an increase in the number of IBA1-positive microglial cells and the upregulation of CD68, a marker for reactive microglia (Kettenmann et al., 2011). We observed an effect of DFP in the number of microglia in regions CA1, CA3, DG, AMY, and PC, wherein DFP + VEH had a significant increase compared to the control (Figure 4D). In regions CA3 and DG, DFP + MPO microglia counts were significantly greater than PBS + MPO (Figure 4D). An evaluation of all brain regions quantified as a whole revealed a main group effect of DFP in both VEH and MPO groups compared to control and PBS + MPO groups, respectively (Figure 4E). At a dose of 10 mg/kg, MPO did reduce the number of microglia in these regions, but it was not statistically significant (Figures 4D, E). Animals that were challenged with DFP had a significant upregulation of CD68-positive microglia (Figure 5). While MPO reduced the mean number of CD68-positive microglial cells in the PC of rats exposed to DFP, there were no significant differences in regions CA1, CA3, DG, AMY, or PC regions (Figures 5B, C).

### MPO had a minimal effect on DFP-induced astrogliosis and no impact on glial scars

**FIGURE 6:**
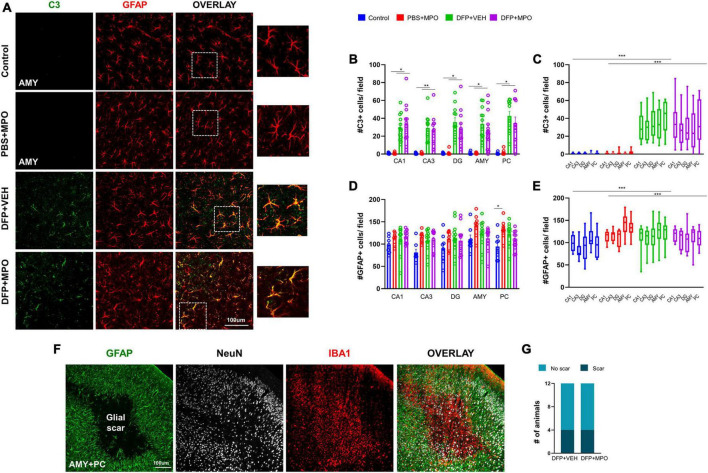
Astrogliosis 1-week post-DFP. **(A)** Representative IHC images of C3 positive (green) astroglia (red) in the amygdala (AMY). **(B)** There was an increase in C3 GFAP-positive cells in CA1, CA3, DG, Amy, and PC in DFP-exposed animals compared to respective controls. Kruskal–Wallis test. **(C)** In grouped hippocampal and extrahippocampal regions, there was a significant upregulation of C3 + GFAP colocalization in DFP + VEH and DFP + MPO compared to control and PBS + MPO groups. Two-way ANOVA mixed effect analysis. **(D)** As determined by the Kruskal–Wallis test (CA1, CA3, and AMY) and one-way ANOVA (DG and PC), DFP nor MPO had an effect on absolute GFAP-positive cell counts in the individual brain regions except for the PC, where there was a significant increase in PBS + MPO animals. **(E)** In contrast, there was a significant difference in the brain as a whole between control vs. DFP + VEH and PBS + MPO vs. DFP + MPO determined by two-way ANOVA mixed effect analysis. MPO had no effect on DFP-induced astrogliosis (*n* = 8 in non-DFP groups, *n* = 12 in DFP groups). **(F)** An example of a glial scar, identified by astrocytes (green) lining clusters of microglia (red), and reduced neuronal cells (white) at the core of the scar. **(G)** The number of animals with glial scars in DFP + VEH and DFP + MPO were compared. *p < 0.05, **p < 0.01, ***p < 0.001.

Astrogliosis was identified as increased GFAP-positive cells and colocalization with complement 3 (C3), a marker for reactive astrocytes (Liddelow et al., 2017; Li et al., 2020). Both were significantly increased in animals challenged with DFP in CA1, CA3, DG, AMY, and PC regions (Figure 6). The DFP + MPO group had reduced the number of C3 and GFAP colocalization in the CA3, DG, AMY, and PC regions when compared to the DFP + VEH group (Figures 6B, C). However, the reductions were not statistically significant. One week of 10 mg/kg MPO treatment, on its own without DFP exposure, did not affect the colocalization of C3 and GFAP in astroglia (Figures 6B, C). When the overall main effect of MPO treatment in DFP animals across the quantified brain regions, MPO did not mitigate DFP-induced astrogliosis (Figures 6C, E). Interestingly, MPO treatment on its own, without DFP, significantly increased GFAP absolute count in the PC (Figure 6D). We have recently discovered the occurrence of glial scars in the amygdala and piriform cortex of animals exposed to DFP (Gage et al., 2022a). In this study, we also found glial scars filled with IBA1-positive cells in both VEH and MPO-treated DFP-exposed groups (Figures 6F, G).

### The effects of MPO treatment on neurodegeneration

**FIGURE 7:**
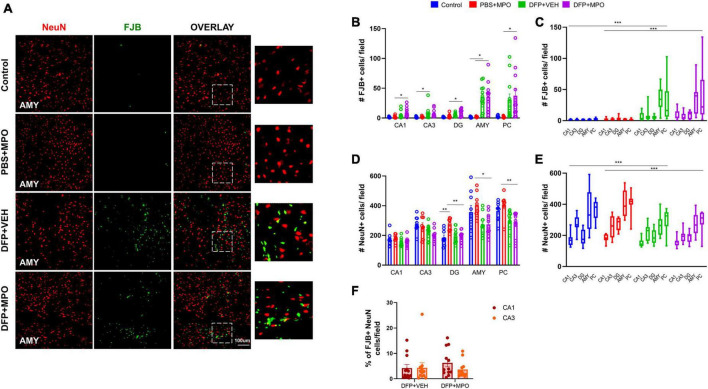
Neurodegeneration 1-week post-DFP. **(A)** Representative images of neurodegeneration demonstrated by FJB-positive (green) NeuN (red) in the amygdala (AMY) of each treatment group. **(B,C)** Cell quantification of FJB and NeuN colocalization in individual and grouped hippocampal (CA1, CA3, and DG), and extrahippocampal (AMY and PC) regions. FJB-positive NeuN cells significantly increased in both DFP + VEH and DFP + MPO groups. One-way ANOVA (CA1, CA3, DG, and AMY), Kruskal–Wallis (PC), and two-way ANOVA mixed effect analysis (grouped regions). **(D,E)** The number of NeuN + cells per field in individual and grouped regions CA1, CA3, DG, AMY, and PC. **(D)** Individual regions were analyzed by one-way ANOVA (CA1, CA3, DG, and AMY) and Kruskal–Wallis (PC). In the DG, AMY, and PC, there was significant neuronal loss in DFP + MPO as compared to PBS + MPO animals. In the DG, PBS + MPO-treated animals had significantly more neurons than controls. **(E)** A two-way ANOVA mixed effect analysis was used to evaluate the grouped regions. Bars indicate a significant difference between control vs. DFP + VEH and PBS + MPO vs. DFP + MPO. **(F)** The percentage of FJB + neurons in CA1 compared to CA3 in DFP + VEH and DFP + MPO animals. No significant differences were observed between groups. *n* = 8 in non-DFP groups, *n* = 12 in DFP groups. *p < 0.05, **p < 0.01, ***p < 0.001.

Neuronal death due to OP exposure was detected by measuring FJB co-stained neurons and the number of NeuN-positive cells (Figure 7A). In CA1, CA3, DG, AMY, and PC, there was a significant increase in FJB-positive neurons in both DFP + VEH and DFP + MPO treated groups (Figures 7B, C). The difference in FJB-positive neurons was noticeable in extrahippocampal regions (Figure 7B). DFP-exposed animals exhibited significant neuronal loss in both hippocampal and extrahippocampal regions of interest. However, this effect was not altered in the MPO group (Figures 7C–E). In oxidative stress models, the CA1 region has previously been shown to be selectively vulnerable to neurodegeneration compared to the CA3 region (Wang and Michaelis, 2010). To account for regional differences in neuronal density, the ratio of FJB-positive cells and NeuN-positive cells was compared in CA1 and CA3 of DFP-exposed animals. We found no significant difference in FJB + NeuN between CA1 and CA3 in the DFP + VEH group (Figure 7F).

## Discussion

We have previously shown that DFP-induced SE increases oxidative stress and exacerbates brain pathology. NOX inhibitors such as DPO (300 mg/kg), an apocynin-derivative, significantly mitigated the DFP-induced effects in a rat DFP model (Putra et al., 2020a). In this study, we hypothesized that a mitochondrial-targeted NOX inhibitor, mitoapocynin (MPO), will yield better outcomes at a lower dose by reducing NOX production of free radicals and, further, oxidative stress in the periphery and the brain due to DFP exposure. Based on the serum kinetics of MPO (10 mg/kg, oral), we tested a treatment regimen of twice a day for the first 3 days, followed by once a day for the next 4 days in a rat DFP model. This dosing regimen reduced DFP-induced oxidative stress and inflammatory markers in the periphery (serum) but had a limited effect in preventing DFP-induced neurodegeneration and gliosis in the brain. Pharmacokinetic studies confirmed that MPO crossed the blood-brain-barrier in a mouse model (Ghosh et al., 2016). In a rat model, we detected 18 ± 9 pg/g MPO in the brain 3 h post-treatment with 10 mg/kg in 2% ethanol. Since MPO is a mitochondrial-targeted drug, we hypothesized that such low brain concentrations of MPO may be enough to mitigate the DFP-induced brain pathology. However, the results did not support the hypothesis. A better formulation and higher concentrations of MPO may yield better results.

DFP, a potent OP, is a surrogate for nerve agents. Nerve agents such as VX and sarin have been used in chemical warfare and terrorist attacks (Chai et al., 2017). The current MCMs effectively prevent nerve agent-induced acute symptoms and mortality only if administered within <20 min of exposure, which is impractical. Importantly, no effective countermeasures are available to mitigate the long-term effects of brain injury in survivors. Nerve agent exposure induces oxidative stress and long-term behavioral deficits in exposed subjects (Gao et al., 2016; Spampanato et al., 2019). Long-lasting neurodegeneration, neuroinflammation, cognitive dysfunction, and other comorbidities will severely impact the quality of life (Okumura et al., 1996; Miyaki et al., 2005). Therefore, preventing the long-term effects of brain injury after exposure to OP nerve agents is an unmet obligation.

Acute exposure to DFP induces SE within minutes of exposure (Rao et al., 2022). SE severity, measured by the number of minutes spent in SE, predicts long-term brain injury and peripheral pathology. Animals that spent <20 min in CS (≥stage 3) during SE presented with moderate gliosis and neurodegeneration in discrete brain regions compared to those that spent > 20 min in CS, in which the brain damage was more widespread (Gage et al., 2021). Therefore, animals must maintain sustained SE severity for more than 20 min to investigate the real therapeutic effects of an interventional drug. Furthermore, it is necessary to balance SE severity between treatment groups (vehicle vs. test drug) to determine the mitigating effects of a test drug. In this study, we achieved SE severity > 30 min and balanced SE severity between vehicle and MPO groups in the age-matched mixed-sex cohort, thus eliminating the potential SE severity variable.

Oxidative and nitroxidative stress, induced by excess ROS and reactive nitrogen species (RNS), occur concurrently and serve as peripheral biomarkers (Davies et al., 2003; Cipak Gasparovic et al., 2017). We found significantly increased serum nitrite, ROS, and GSSG levels at 8 days post-DFP challenge. Treating with MPO significantly reduced nitrite concentrations in DFP-challenged animals. MPO also reduced increased ROS levels in DFP-exposed animals. High oxidized glutathione (GSSG) levels imply increased scavenging of excess ROS by glutathione (Owen and Butterfield, 2010). The significant reduction in GSSG in MPO-treated DFP-challenged animals suggests rescue of the altered redox potential. In our previous DFP studies, increased serum nitrite levels were also observed at 6 weeks post-exposure (Putra et al., 2020a,b). DPO treatment significantly reduced DFP-induced nitrite levels (Putra et al., 2020a). These findings demonstrate the beneficial effects of MPO against DFP-induced oxidative stress in the peripheral circulation.

OP-induced nitroxidative or oxidative stress can promote proinflammatory cytokines or vice versa. For example, RNS activation of TNF-α via the NFkB signaling pathway promotes excessive generation of ROS (Davies et al., 2003; Yang et al., 2018). Therefore, it is unsurprising that DFP exposure significantly increased proinflammatory cytokines IL-1β, IL-6, and TNF-α. Previous studies reported similar findings in the DFP model (Putra et al., 2020b). In this study, we demonstrated a significant reduction of these cytokines in MPO-treated animals exposed to DFP. Neither DFP nor MPO significantly altered IL-10 or MCP1 levels in the serum. This is likely attributable to the functionality of cytokine IL-10 as both anti- and proinflammatory, and MCP1, which is a monocyte chemoattractant produced by circulating monocytes and astrocytes (Gao et al., 2009; Yadav et al., 2010).

Microglia, the resident macrophages of the brain, are known to proliferate after exposure to OP nerve agents (Kettenmann et al., 2011; Gage et al., 2022a). Activated/reactive microglia can become maladaptive and release excess amounts of ROS, RNS, and cytokines. Additionally, they may change in morphology indicative of a phagocytic nature (Lee et al., 2021). The Cluster of Differentiation 68 (CD68) is a lysosomal transmembrane glycoprotein expressed in high levels in phagocytic microglia and widely used as a marker for reactive microglia (Kettenmann et al., 2011). In our DFP model, CD68 expressing microglia were significantly increased in both DFP + VEH and DFP + MPO groups in the hippocampus, amygdala, and piriform cortex. MPO marginally reduced the presence of microglia in extrahippocampal and hippocampal regions and CD68 in the PC in DFP-challenged animals. Interestingly, we did not observe a reduction in DFP-induced microgliosis or CD68-positive microglia in DPO-treated animals in the 6-week DFP study (Putra et al., 2020a). It is likely that MPO may attenuate microgliosis at a higher dose and could produce a potent anti-inflammatory effect in the brain.

We investigated NOX2 because of its role in neuroinflammation and high expression in microglial cells (Ma et al., 2017). At rest, NOX2 has both cytosolic and membrane-bound subunits. In an *in vitro* study, MPO treatment of cultured microglia cell lines restricted the translocation of the cytosolic subunits of NOX to its membrane-bound subunits, preventing enzyme activation and the production of free radicals (Langley et al., 2017). Following brain injury, its cytosolic subunits translocate to membrane-bound GP91*^phox^* to form a functional complex, initiating free radical production by NOX2 (Gao et al., 2012; Ma et al., 2017). Similar to our previous 6-week DFP study (Putra et al., 2020a), we observed a significant increase in the number of GP91*^phox^*-positive cells in the brain as early as 1-week post-exposure in this study. While increased GP91*^phox^* level is associated with ROS production, its presence alone does not necessarily indicate NOX activation (Ma et al., 2017). A limitation of the current study is the lack of investigation of cytosolic subunits translocation to the membrane, which is challenging in an *in vivo* model. Other NOX enzymes may likely be involved in DFP-induced toxicity, and NOX inhibitors DPO and MPO may also suppress ROS production through these pathways, which is not invested in this study. Astroglia expresses NOX1, NOX4, or both (Gao et al., 2012). An increase in astrocytes may imply the upregulation of NOX and ROS production (Chen et al., 2020). Future studies will investigate the direct effect of MPO on the astroglial NOX1 and NOX4 responses to DFP. Astrogliosis, as such, is not necessarily harmful; however, complement 3 (C3) expression by astrocytes may suggest their reactivity in response to an injury (Wei et al., 2021). C3 is a mediator of innate immunity and interacts with microglia, and promotes synaptic pruning (Stevens et al., 2007; Shinjyo et al., 2009). We observed a significant increase in reactive astrogliosis in previous DFP studies at 8 days and 6 weeks post-DFP exposure (Putra et al., 2020a,b). DPO significantly mitigated both reactive astrogliosis and C3-positive astrocytes in a 6-week DFP study (Putra et al., 2020a). In the present study, we did observe a significant increase in C3-positive astrocytes in hippocampal and extrahippocampal brain regions of the DFP-exposed vehicle-treated group, but MPO had no effect. In the PC, there was an unexpectedly significant increase of C3-positive astroglia in non-DFP animals. In an *in vitro* manganese exposure model, MPO reduced the astroglial inflammatory response by reducing IL-1β and IL-18 expression (Sarkar et al., 2017). We have shown a reduction of C3 in a DFP model with a high dose of DPO (300 mg/kg) (Putra et al., 2020a). Therefore, the prospect of a differential impact of MPO on NOX or cell-specific ROS generation in a dose-dependent manner requires further investigation.

Cortical glial scars in the DFP model are characterized by reactive astroglia lining the scar with clusters of reactive microglia and degenerating neurons at its core (Gage et al., 2022a). In our study, one-third of animals with glial scars were observed in DFP + VEH and DFP + MPO groups. MPO at 10 mg/kg did not impact the occurrence of glial scars in this study. In DFP-exposed animals, neurodegeneration was observed in the hippocampus and was especially distinct in the amygdala and piriform cortex, where glial scars typically occur in DFP models. In a previous study, neurons in the CA1 region were found to be vulnerable to cell death and had a higher mitochondrial production of ROS than neurons in the CA3 region (Wang and Michaelis, 2010). In DFP + MPO-treated animals in this study, we found a trend in this direction; however, it did not reach statistical significance. MPO at 10 mg/kg did not reduce DFP-induced neurodegeneration. A dose optimization study is required in future investigations.

Overall, DFP exposure significantly increased oxidative stress and proinflammatory cytokines in the serum and propagated reactive microgliosis and astrogliosis, and neurodegeneration in key brain regions. MPO attenuated DFP-induced oxidative stress and proinflammatory cytokines in peripheral circulation but only minimally reduced reactive microgliosis and astrogliosis in the brain. Considering the known pharmacokinetic challenges of apocynin and DPO, MPO also revealed bioavailability concerns, which are under further investigation. Our study provides evidence that at the dose of 10 mg/kg, MPO is not effective in the brain despite its ability to cross the blood-brain-barrier. Optimization of the dosing regimen and drug formulation is required to determine the efficacy of MPO in the DFP model.

## Data availability statement

The original contributions presented in this study are included in this article/supplementary material, further inquiries can be directed to the corresponding author.

## Ethics statement

The animal study was reviewed and approved by the Iowa State University IACUC committee.

## Author contributions

TT conceptualized, designed and supervised the experiments, secured the funding, and edited the manuscript. CM conducted the experiments, acquired and analyzed the data, and drafted the manuscript. NR, SV, MG, and MP assisted the experiments, compiled and cross-verified the data, and proofread the manuscript. BP conducted the IHC and image analysis. CH and JH contributed to the cell quantification. All authors contributed to the article and approved the submitted version.
